# The generic version of China Health Related Outcomes Measures (CHROME-G): psychometric testing and comparative performance with the EQ-5D-5L and SF-6Dv2 among the Chinese general population

**DOI:** 10.1186/s12889-024-20999-4

**Published:** 2024-12-18

**Authors:** Shitong Xie, Jing Wu, Pinan Chen, Xiaoning He, Kun Zhao, Feng Xie, for the CHROME Study Group

**Affiliations:** 1 School of Pharmaceutical Science and Technology, Faculty of Medicine, Tianjin University, Tianjin, China; 2 Center for Social Science Survey and Data, Tianjin University, Tianjin, China; 3 Vanke School of Public Health, Tsinghua University, Beijing, China; 4 Department of Health Research Methods, Evidence and Impact, McMaster University, Hamilton, ON Canada; 5 Centre for Health Economics and Policy Analysis, McMaster University, Hamilton, ON Canada

**Keywords:** CHROME-G, Psychometric property, EQ-5D-5L, SF-6Dv2, Health-related quality of life

## Abstract

**Objectives:**

The CHROME-G is the first generic preference-based measure developed in China. This study aimed to validate and compare the psychometric properties of the CHROME-G with the EQ-5D-5L and SF-6Dv2 among the Chinese general population.

**Methods:**

A representative sample of the Chinese general population in terms of age, gender, education, and urban/rural residence was recruited for an online survey. During the survey, respondents completed three instruments (first the CHROME-G, then the EQ-5D-5L and SF-6Dv2 in random order), demographic and health-related questions. The retest survey was carried out after two weeks. Ceiling/floor effects were first assessed. Convergent and divergent validity was examined using Spearman’s rank correlation. Known-group validity was examined using the non-parametric Kruskal–Wallis H test and effect size. Test–retest reliability was assessed using the intraclass correlation coefficient and weighted Kappa statistics.

**Results:**

One thousand respondents (51.1% male, mean age 44.7 years) completed the first survey, with 378 also completing the retest survey. The mean ± SD completion time was 2.03 ± 0.58 min for the CHROME-G, and 1.37 ± 0.54 and 1.13 ± 0.38 min for the EQ-5D-5L and SF-6Dv2. Only the EQ-5D-5L had a ceiling effect of 35.1%. The range of Spearman rank’s correlations was 0.45–0.62 for convergent validity and 0.14–0.46 for divergent validity. Among different health subgroups, the effect size for the CHROME-G, EQ-5D-5L and SF-6Dv2 was 1.348–3.416, 1.362–3.325 and 1.097–2.228, respectively. The ICC for test–retest was 0.791 for the CHROME-G, compared with 0.994 and 0.971 for the EQ-5D-5L and SF-6Dv2.

**Conclusions:**

The CHROME-G showed good and comparable psychometric properties with the EQ-5D-5L and SF-6Dv2.

**Supplementary Information:**

The online version contains supplementary material available at 10.1186/s12889-024-20999-4.

## Background

The latest edition of China Economic Evaluation Guidelines published in 2020 recommends the use of generic preference-based measures (GPBMs), similar to many other countries’ recommendations [1, 2]. Commonly used GPBMs include the EQ-5D and SF-6D, which were originally developed in Western countries [3, 4]. These instruments have been translated into Chinese and validated in general and patient populations [5–15]. Recently, Chinese-specific value sets were also developed for these instruments [5, 6, 16, 17], which facilitates their use in health technology assessments, randomized clinical trials and population health surveys in China.

However, there are growing concerns about the cultural equivalence of existing GPBMs as perceptions and preferences for health differ in China [18–24]. Empirical evidence has shown that most Chinese in the general public are not concerned about self-care, while anxiety or depression, as phrased in the EQ-5D is not well understood by many with low education levels [19, 23]. More importantly, existing GPBMs may not include dimensions perceived as important by the Chinese population, such as appetite and sleep [19, 21, 22, 24]. Empirical evidence has shown that the EQ-5D suffered from a ceiling effect, while the SF-6D showed a slight floor effect [13–15, 25–27]. The China Health Related Outcomes Measures (CHROME) was an initiative aimed at developing a series of health-related quality of life (HRQoL) instruments specifically for Chinese populations. The generic version of the CHROME (CHROME-G) has been developed, which was reported elsewhere [28]. The objective of this study was to validate the CHROME-G and compare its performance with the EQ-5D-5L and SF-6Dv2 in the Chinese general population.

## Methods

We conducted a national online survey between December 2021 and January 2022. The study protocol was approved by the Academic Ethics Committee at Tianjin University (Reference No. TJUE-2021–168), and all respondents provided informed consent.

### Respondents

Respondents were recruited through online survey panels. The panels sent text messages, emails, and push notification within apps to eligible panel members asking if they would like to participate in the survey. Eligible respondents had to meet the following inclusion criteria: 1) over 18 years of age; 2) having Chinese nationality; 3) having lived in mainland China for the past five years; and 4) having good cognitive ability.

Rules of thumb, previous reviews, and COSMIN recommendation suggests that the sample size for a quantitative instrument evaluation study should be at least 10 times the number of items to be analysed, or between 100 and 500 [29–31]. Taking into account the large size of population in China, the target sample size for this survey was set at 1000 (two times of 500). Sampling quota was stratified by age, gender, education, urban/rural residence, and region of residence (northeast, east, north, central, south, southwest and northwest), reflecting the distribution of the key characteristics of the general Chinese population.

According to the sample size formula for the critical indicator of test–retest reliability, namely the intra-class correlation coefficient (ICC), at least 160 respondents was required for the CHROME-G, which consists of 12 items, assuming a planned ICC value of 0.7, a two-sided 95% confidence interval, and a desired width of 0.1 [32]. In contrast, both the EQ-5D-5L with five questions and SF-6Dv2 with six questions necessitate fewer samples than the CHROME-G. Consequently, the target sample size for the retest survey was established at 320 (two times of 160), with sampling quotas stratified according to the same characteristics as those used in the initial survey. Appendix Table 1 outlines the sample requirements for each quota in both surveys.

### Instruments

The CHROME-G comprises 12 items (refer to unique one question for each item) measuring pain, fatigue, appetite, sleeping, vision, hearing, memory, mobility, daily activities, mood, worry, and social interaction [28]. The items appetite, sleeping, mobility and daily activities have five levels (response options), while all others have four levels. The levels are listed in ascending order of severity under each question. This instrument uses “the past seven days” as the recall period. More detailed information can be found elsewhere [28].

The EQ-5D-5L comprises five dimensions, including mobility, self-care, usual activities, pain/discomfort, and anxiety/depression, with one question for each dimension. Each question has five levels (response options), listed from least to most severe, e.g. “I have no/ slight/ moderate/ severe/ extreme pain or discomfort”. The EQ-5D-5L uses the recall period of “today”. The EQ-5D-5L also includes a vertical visual analog scale (VAS), i.e., the EQ VAS, aiming to measure self-rated health status ranging from 0 (worst imaginable health state) to 100 (best imaginable health state) [33].

The SF-6Dv2 is derived from 10 items of the second version of the 36-item Short Form Health Survey (SF-36v2). There are six dimensions, which also equates to six questions, related to physical functioning, role limitation, social functioning, pain, mental health, and vitality. All questions have five levels (response options), except for pain, which has six levels. Both the questions of physical functioning and pain have intensity levels, listed from least to most severe; while the other four questions have frequency levels, e.g. “social activities are limited none/ a little/ some/ most/ all of the time”. A recall period of “the last four weeks” is used for the SF-6Dv2 [34].

Validated Chinese versions of the EQ-5D-5L and SF-6Dv2 are available [6, 7]. The study has obtained approval for these Chinese versions from the institutions that developed the instruments.

### Data collection

Data were collected through a self-completed online survey via mobile phone or computer. Before the start of the survey, each respondent was asked a few screening questions to ensure the quota and inclusion criteria were met. Once the eligibility was confirmed, respondents were informed of the purpose and content of the survey through an informed consent. They would also be reminded to complete the questionnaire in person through the informed consent and pop-up windows (at the beginning of each section of the questionnaire).

Respondents were first asked to complete the CHROME-G and provide feedback on their difficulties in understanding and completing the instrument. Respondents then completed the EQ-5D-5L and SF-6Dv2 in a random order. The time taken by respondents to complete each of the three instruments was recorded. Finally, respondents answered demographic questions (including ethnicity, dialect, hukou, marital status, residence, number of family members, employment status, personal monthly income, and health insurance) and health-related questions (including height and weight, self-reported health status, self-reported chronic diseases, smoking and alcohol consumption, self-reported health level, and life satisfaction).

After two weeks, respondents received the link to the retest questionnaire and chose whether or not to continue. Screening questions for the retest were: “Have there been any changes in your health status since the last time you completed the survey?” (rated on a 5-level Likert scale “no change”, “slightly change”, “some change”, “much change”, or “extremely change”) and “How would you rate your current health status?” (rated on a 5-level Likert scale “very good”, “good”, “fair”, “poor”, or “very poor”). Only those with “no change” or “slightly change” to the first question and whose health status has not been changed or were at adjacent levels between the two times could be included. In the retest survey, respondents completed the CHROME-G, EQ-5D-5L and SF-6Dv2 in the same order as in the first survey.

Quality control was applied to ensure the data quality and respondents were excluded if 1) they completed each instrument in less than five seconds; and (2) three records were identified under the same IP address. Fifteen respondents participated in the pilot test to identify any problems with the online survey.

### Statistical analysis

We followed general recommendations for the design of instrument validations in the COSMIN checklist [31]. Respondent characteristics were described using descriptive statistics, including mean, standard deviation [SD], number, or proportion. The mean time to complete the three instruments was calculated. The utility values of the EQ-5D-5L and SF-6Dv2 were calculated using the corresponding utility value sets based on the general Chinese population [6, 7] to describe the overall health of respondents.

Given that the CHROME-G has not yet developed a utility value set, we utilized level sum scores rather than utility values to facilitate comparisons of the psychometric properties between the CHROME-G and both the EQ-5D-5L and SF-6Dv2. For all three instruments, higher scores indicate poorer health. The response distribution and the level sum score were plotted for the three instruments. The ceiling and floor and effects for each measure were assessed by examining the percentage of respondents in the best and worst health states, respectively. These effects are considered to exist if more than 15% of the respondents achieved either extreme end of the scale [35].

Convergent validity was assessed between conceptually similar items of the CHROME-G, the EQ-5D-5L and the SF-6Dv2, whereas divergent validity was assessed between conceptually different items of these instruments. We hypothesized strong correlations for similar conceptual items and moderate to weaker correlations for different conceptual items (see detailed hypotheses in Appendix Table 2). Spearman’s rank correlation coefficients (r) demonstrate the strength of the correlation: strong (r ≥ 0.5), moderate (0.35 ≤ r < 0.5), weak (0.2 ≤ r < 0.35), and poor (r < 0.2) [36].

Known-group validity confirmed the hypothesized differences between subgroups that are known to be different. Based on the published literature [13, 14], the study hypothesized that respondents with poorer self-reported health status or more chronic diseases would have higher level sum scores. The mean level sum score of each subgroup of self-reported health status, EQ-VAS scores, and number of self-reported chronic diseases was calculated. The non-parametric Kruskal–Wallis H test was used to analyze significant differences and the effect size was calculated. For polytomous variables, the effect size between the extreme subgroups (e.g., self-reported very good health state subgroup and self-reported very poor health state subgroup) was calculated. Cohen’s criteria define an effect size between 0.2 and 0.5 as small, between 0.5 and 0.8 as moderate, and more than 0.8 as large [37].

For test–retest reliability, the intra-class correlation coefficient (ICC) and standard error of measurement (SEM) on the level sum score were calculated using the two-way mixed effects model based on absolute agreement [38]. An ICC value greater than 0.7 is considered satisfactory [36]. For each of the items, the percentage of actual agreement and weighted Kappa statistics were calculated. Weighted Kappa statistics indicate 0.81–1.00 as almost perfect agreement, 0.61–0.80 as substantial, 0.41–0.60 as moderate, 0.21–0.40 as fair, 0.01–0.20 as none to slight, and ≤ 0 as no agreement [39].

The statistical analysis was conducted using STATA 17.0 (StataCorp LLC, College Station, TX, USA). All reported statistical tests were performed two-sided with a significance level of 0.05 unless otherwise stated.

## Results

### Respondent characteristics

Three thousand seventy-eight people were invited to participate in the survey. After excluding refusals, mid-round opt-outs, and those who did not meet the quota and quality control requirements, a total of 1000 respondents with valid data were included in the analysis (Appendix Table 3). The mean (SD) duration of the whole survey was 10.18 (2.52) minutes. Most respondents (767 [76.70%]) completed the online survey by telephone, with the remaining using the computer.

Respondents came from 239 cities in 31 provinces across mainland China, with a diverse geographical distribution as shown in Appendix Fig. 1a. As seen in Table 1, 51.1% were male; the mean (SD) age was 44.69 (14.79) years, with a range from 18 to 73 years. The characteristics of the respondents were representative of the general population in China. The mean (SD) utility value among all respondents was 0.891 (0.143) for the EQ-5D-5L and 0.697 (0.195) for the SF-6Dv2 (Appendix Table 4).

**Table 1:** The characteristics of respondents

Characteristics	Total sample (*N* = 1000) N (%)	Retest sample (*N* = 378) N (%)	Chinese general population (%)^a^
***Gender***^***b***^			
Male	511 (51.1%)	206 (54.5%)	51.1%
Female	489 (48.9%)	172 (45.5%)	48.9%
***Age, mean* ± *SD***	44.69 ± 14.79	47.77 ± 13.34	N/A
***Age group, years***^***b***^			
18–29	189 (18.9%)	45 (11.9%)	18.9%
30–39	195 (19.5%)	49 (13.0%)	19.6%
40–49	199 (19.9%)	95 (25.1%)	19.8%
50–59	191 (19.1%)	96 (25.4%)	19.1%
≥ 60	226 (22.6%)	93 (24.6%)	22.6%
***Education***^***b***^			
Primary or lower	269 (26.9%)	109 (28.8%)	26.9%
Junior high school	390 (39.0%)	165 (43.7%)	39.1%
Senior high school	175 (17.5%)	60 (15.9%)	17.2%
College or higher	166 (16.6%)	44 (11.6%)	16.8%
***Residence***^***b***^			
Urban	606 (60.6%)	217 (57.4%)	60.6%
Rural	394 (39.4%)	161 (42.6%)	39.4%
***Region***^***b***^			
Northeast	80 (8.0%)	36 (9.5%)	7.7%
East	300 (30.0%)	110 (29.1%)	29.5%
North	120 (12.0%)	42 (11.1%)	12.5%
Central	160 (16.0%)	56 (14.8%)	16.0%
South	120 (12.0%)	44 (11.7%)	12.4%
Southwest	150 (15.0%)	56 (14.8%)	14.5%
Northwest	70 (7.0%)	34 (9.0%)	7.4%
***Marital status***			
Unmarried	189 (18.9%)	45 (11.9%)	18.0%
Married	779 (77.9%)	316 (83.6%)	74.0%
Divorced	22 (2.2%)	9 (2.4%)	2.3%
Widowed	10 (1.0%)	8 (2.1%)	5.7%
***Employment status***			
Employed	717 (71.7%)	264 (69.8%)	N/A
Retired	185 (18.5%)	78 (20.6%)	N/A
Student	29 (2.9%)	4 (1.1%)	N/A
Unemployed	69 (6.9%)	32 (8.5%)	N/A
***Personal monthly income (in RMB)***			
< ¥2000	109 (10.9%)	41 (10.8%)	N/A
¥2000 ~ 5000	344 (34.4%)	124 (32.8%)	N/A
¥5000 ~ 10,000	411 (41.1%)	171 (45.3%)	N/A
> ¥10,000	136 (13.6%)	42 (11.1%)	N/A
***Number of self-reported chronic diseases***^***c***^			
0	571 (57.1%)	207 (54.8%)	N/A
1	183 (18.3%)	73 (19.3%)	N/A
2	125 (12.5%)	50 (13.2%)	N/A
≥ 3	121 (12.1%)	37 (12.7%)	N/A
***Self-reported health status***			
Very good	169 (16.9%)	31 (8.2%)	N/A
Good	407 (40.7%)	213 (56.3%)	N/A
Fair	362 (36.2%)	122 (32.3%)	N/A
Poor	56 (5.6%)	12 (3.2%)	N/A
Very poor	6 (0.6%)	0 (0.0%)	N/A

^a^Statistics data of the Chinese general population were extracted from the *China Statistical Yearbook (2020)*. When the statistical scale of the original data was not calculated as the general population aged ≥ 18 years, the data were adjusted based on the proportion of the population of each age to the total population in this study. N/A indicates that data was not included in the public available data source

^b^The quota sampling was used in this study, which five quotas, i.e., gender, age, education, urban/rural of residence, and region of residence, were pre-defined on the basis of their distribution in the Chinese general population

^c^Chronic diseases include hypertension, dyslipidemia, diabetes or high blood sugar, brain diseases, thyroid conditions, sense conditions (e.g. glaucoma, otitis, rhinitis, etc.), stroke, osteoporosis, heart diseases, liver or gallbladder diseases, kidney diseases, lung diseases, stomach or other digestive diseases, urinary system disease, arthritis or rheumatism, cancer or malignant tumor, anemia or blood disorders, skin disorders, emotional or psychiatric problems, or other respondent-reported chronic diseases

### Measurement properties of the CHROME-G, EQ-5D-5L and SF-6Dv2

#### Time to completion

The mean (SD) time for completing the CHROME-G, EQ-5D-5L and SF-6Dv2 were 2.03 (0.58) minutes, 1.37 (0.54) minutes, and 1.13 (0.38) minutes, respectively. 97.1% of the respondents rated the CHROME-G as “very easy” or “easy” or “general” to understand and 98.7% rated it as “very easy” or “easy” or “general” to complete. Only less than 5% of the respondents gave a negative rating (“very difficult” or “difficult”) for understanding and answering. Details of the ratings are described in Appendix Table 5.

#### Response distribution

**Fig. 1:**
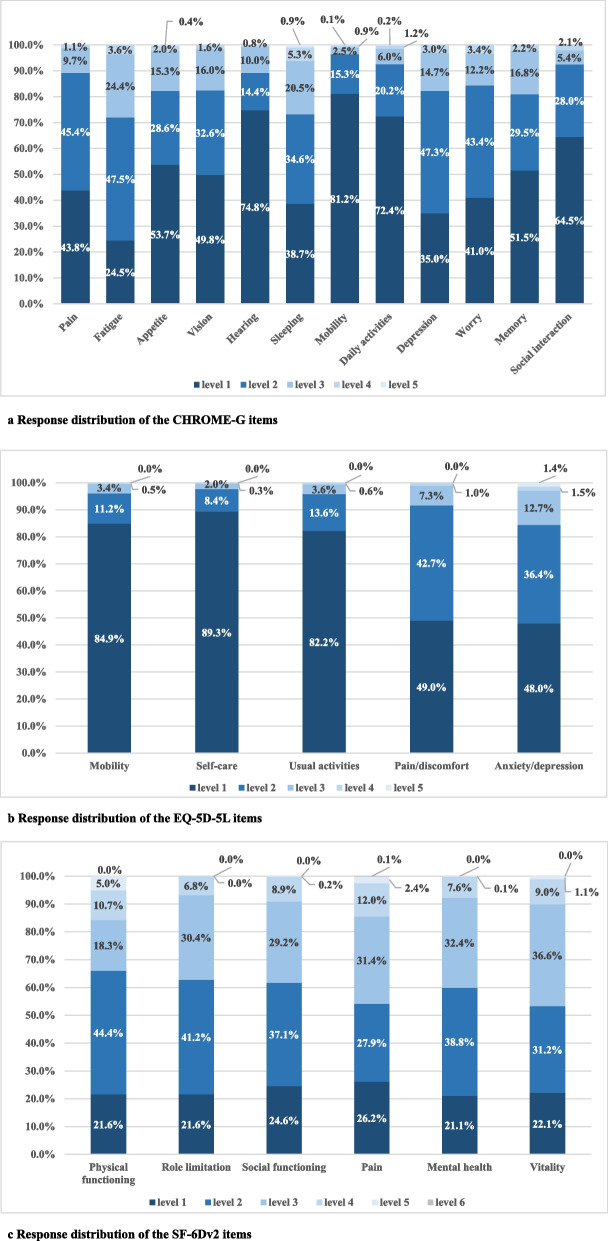
Response distribution of the three instruments (*N* = 1000)

Among the CHROME-G items, fatigue reported the highest proportion (75.5%) of having any problems, followed by mood (65.0%), sleeping (61.3%), worry (59.0%), pain (56.2%), and vision (50.2%); other six items reported less than 50% of any problems, that is, memory (48.5%), appetite (46.3%), social interactions (35.5%), daily activities (27.6%), hearing (25.2%), and mobility (18.8%) (Fig. 1a). For the EQ-5D-5L, anxiety/depression reported the highest proportion (52.0%) of having any problems, followed by pain/discomfort (51.0%), usual activities (17.8%), mobility (15.1%) and self-care (10.7%) (Fig. 1b). For the SF-6FDv2, mental health reported the highest proportion (78.9%) of having any problems, followed by physical functioning/role limitation (78.4%), vitality (77.9%), social functioning (75.4%) and pain (73.8%) (Fig. 1c).

#### Ceiling and floor effects

As shown in Appendix Fig. 2, the ceiling effect of the CHROME-G (54, 5.4%) and SF-6Dv2 (118, 11.8%) were both acceptable, although the SF-6Dv2 had a higher proportion of respondents reporting the best health state than the CHROME-G. However, an apparent ceiling effect was found in the EQ-5D-5L, with 351 respondents (35.1%) reporting the best health state. There was no floor effect in the CHROME-G, EQ-5D-5L and SF-6Dv2.

#### Convergent and divergent validity

As shown in Table 2, most of the similar conceptual items were strongly correlated, with Spearman’s rank correlation coefficients ranging from 0.45 to 0.62 (*p* < 0.001). Only pain and social interactions in the CHROME-G showed moderate correlations with corresponding items in the SF-6Dv2, being 0.46 and 0.45, respectively. All the different conceptual items were moderately or weakly correlated, with Spearman’s rank correlation coefficients ranging from 0.14 to 0.46 (*p* < 0.001).

**Table 2:**
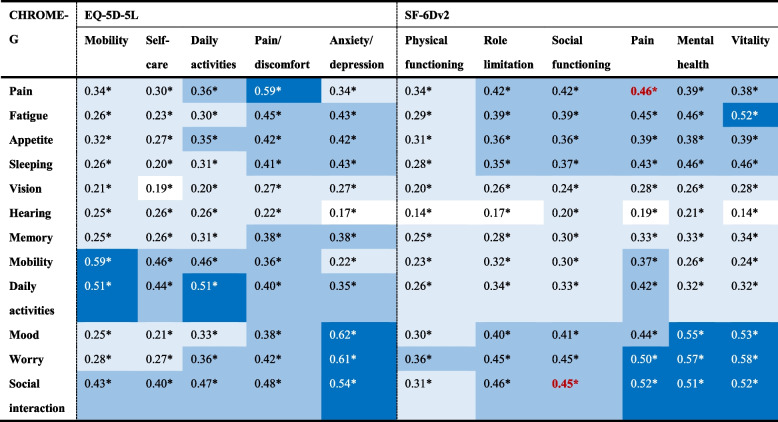
Correlations of the items between the three instruments (*N*=1000)

Spearman’s rank correlation coefficients (r) perform the correlations

Dark blue indicates the strong correlation (r≥0.5), blue indicates the moderate correlation (0.35 ≤r<0.5), light blue indicates the weak correlation (0.2≤r<0.35), and white indicates the poor correlation (r<0.2)

Some items hypothesized to be strongly correlated but only moderately or weakly correlated are marked in red font

*P < 0.001

#### Known-group validity

As shown in Table 3, the level sum scores of the three instruments were all significantly difference (*p* < 0.001) across different health groups classified by self-reported health status, EQ-VAS scores, and number of self-reported chronic diseases. All the effect sizes were large; and the effect size of the CHROME-G (1.348 [95% CI 1.139, 1.556]—3.416 [95% CI 2.521, 4.302]) was similar to the EQ-5D-5L (1.362 [95% CI 1.153, 1.571]—3.325 [95% CI 2.435, 4.208]), but generally larger than the SF-6Dv2 (1.097 [95% CI 0.892, 1.301]—2.228 [95% CI 1.378, 3.072]).

**Table 3:** Known-group validity of the three instruments within different health groups (*N* = 1000)

	**N (%)**	**CHROME-G**		**EQ-5D-5L**		**SF-6Dv2**	
		**Mean (SD)**	**ES (95% CI)**	**Mean (SD)**	**ES (95% CI)**	**Mean (SD)**	**ES (95% CI)**
**Self-reported health states**			3.416 (2.521, 4.302)		3.325 (2.435, 4.208)		2.228 (1.378, 3.072)
Very good	169 (16.9%)	15.94 (4.81)		5.76 (1.92)		9.88 (4.56)	
Good	407 (40.7%)	18.11 (4.03)		6.12 (1.51)		12.84 (4.13)	
Fair	362 (36.2%)	22.15 (4.61)		7.63 (2.05)		15.80 (3.04)	
Poor	56 (5.6%)	27.45 (6.59)		10.23 (2.97)		18.64 (2.94)	
Very poor	6 (0.6%)	33.00 (9.27)		12.5 (4.18)		20.17 (6.34)	
**EQ-VAS score in EQ-5D-5L**			2.410 (2.102, 2.716)		2.712 (2.394, 3.028)		1.846 (1.554, 2.137)
≥ 90	437 (43.7%)	16.83 (4.22)		5.76 (1.58)		11.29 (4.40)	
80–89	289 (28.9%)	20.57 (4.38)		6.96 (1.64)		14.77 (3.14)	
70–79	147 (14.7%)	21.56 (4.56)		7.41 (1.84)		15.38 (3.91)	
60–69	66 (6.6%)	25.00 (5.17)		9.18 (2.49)		17.39 (2.73)	
< 60	61 (6.1%)	27.87 (6.65)		10.66 (2.97)		19.11 (2.75)	
**Number of self-reported chronic diseases**			1.348 (1.139, 1.556)		1.362 (1.153, 1.571)		1.097 (0.892, 1.301)
0	571 (57.1%)	5.75 (1.21)		6.15 (1.86)		12.18 (4.68)	
1	183 (18.3%)	6.51 (1.36)		7.24 (2.16)		15.16 (3.40)	
2	125 (12.5%)	7.03 (1.45)		7.66 (1.97)		15.92 (3.01)	
≥ 3	121 (12.1%)	7.51 (1.75)		8.93 (2.71)		17.03 (2.95)	

Non-parametric Kruskal–Wallis H test indicated that the differences in the level sum score between the self-reported health states, EQ-VAS scores (from the EQ-5D-5L) and number of self-reported chronic diseases of the three instruments are all statistically significant (*p* < 0.001)

*SD* Standard deviation, *ES* Effect size

#### Test–retest reliability

Five hundred ninety-four people were invited and 378 were finally included in the analysis (Appendix Table 3). Retest respondents came from 141 cities in 29 provinces across mainland China (Appendix Fig. 2b). 54.5% of them were male and the mean (SD) age was 47.77 (13.34) years, ranging from 18 to 72 years (Table 1). The mean (SD) utility value among retest respondents was 0.913 (0.096) for the EQ-5D-5L and 0.719 (0.149) for the SF-6Dv2, which were higher than the first survey (Appendix Table 4).

As shown in Table 4, ICC values for the three instruments were all greater than 0.7. Although the agreement between the two surveys of the CHROME-G was good (0.791 [95% CI 0.738, 0.833]), it was not as excellent as that of the EQ-5D-5L (0.994 [95% CI 0.992, 0.995]) and SF-6Dv2 (0.971 [95% CI 0.965, 0.976]). As Table 5 presented, the retest agreement of each item in the CHROME-G, EQ-5D-5L and SF-6Dv2 were 0.980 (vision)−0.258 (pain), 0.963 (mobility)−0.610 (pain/discomfort) and 0.985 (pain)−0.710 (mental health) respectively. For the CHROME-G, social interaction, daily activities and mobility showed almost perfect agreement that was similar to the other two instruments; all the unique items showed perfect agreement (vision, hearing) or moderate agreement (appetite, sleeping, memory); pain, mood, fatigue and worry showed fair agreements and had completely lower weighted Kappa values than similar items in the other two instruments.

**Table 4:** Test–retest reliability of the three instruments (*N* = 378)

	**Level sum score**			**ICC (95% CI)**	**SEM**
	**First survey Mean (SD)**	**Retest survey Mean (SD)**	***P* value**		
CHROME-G	19.30 (4.88)	18.54 (4.17)	< 0.001	0.791^*^ (0.738, 0.833)	2.080
EQ-5D-5L	6.52 (1.86)	6.49 (1.83)	0.013	0.994^*^ (0.992, 0.995)	0.143
SF-6Dv2	13.31 (4.30)	13.31 (4.00)	0.620	0.971^*^ (0.965, 0.976)	0.707

*ICC* Intra-class correlation coefficient, *SEM* Standard error of measurement

^*^P < 0.05

## Discussion

This study provided the first empirical evidence for the psychometric testing of the recently developed CHROME-G and a comparison of its measurement performance with the commonly used EQ-5D-5L and SF-6Dv2. Properties tested and compared included time to completion, response distribution, ceiling and floor effects, convergent and divergent validity, known-group validity and test–retest reliability. This study supports the feasibility of using the CHROME-G among the Chinese population.

**Table 5:**
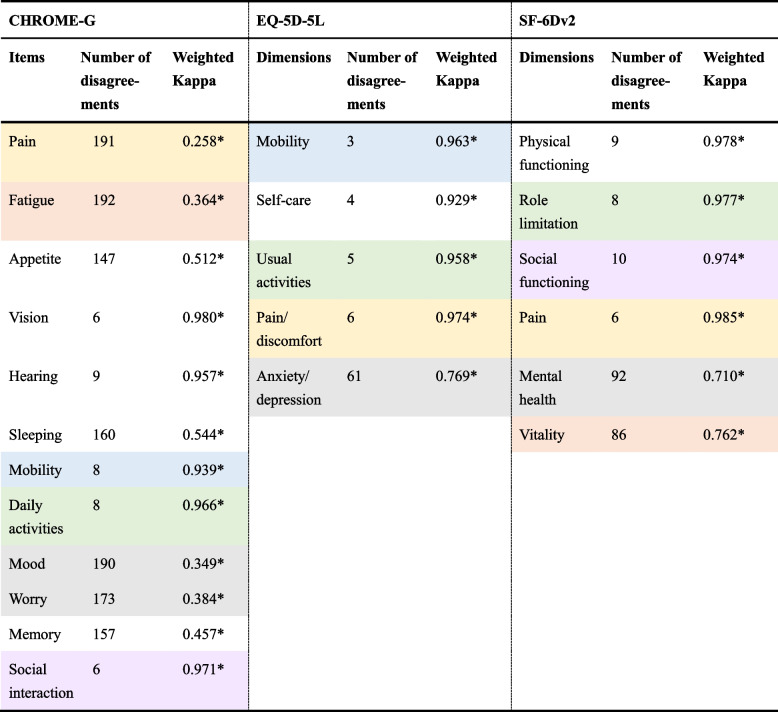
Weighted Kappa statistics of the items in three instruments (*N*=378)

Weighted Kappa statistics not only takes into account agreement, but also the degree of agreement

*P < 0.05

The CHROME-G was considered to have a good acceptability, with a completion time of only 2.03 min (each item took on average about 10 s to complete) and a proportion of understanding/completing difficulties of less than 5%. We attributed this to the good content validity of the instrument. Through directly and extensively capturing the views of the Chinese sample and seeking their input on how health conditions or problems affect HRQoL, the CHROME-G covered 12 HRQoL items perceived as most important by the Chinese. Given the item concepts are fully relevant and the item wordings are highly consistent with the idiomatic expressions, Chinese respondents are capable of completing the CHROME-G easily and quickly.

The response distribution showed that the CHROME-G had a wider range of proportions reporting any health problems than the EQ-5D-5L and SF-6Dv2. The main reason is that the CHROME-G contains more low-severity items (e.g., fatigue and sleeping), which tend to measure relatively mild health problems than the EQ-5D-5L, and contains more high-severity items (e.g., mobility and daily activities), which tend to measure more severe health problems than the SF-6Dv2. Due to the predominance of high-severity items, the EQ-5D-5L presented ceiling effects, consistent with previous studies in the Chinese general population [13, 14]. The study also noticed that the CHROME-G had a lower proportion of reporting the best health state than the SF-6Dv2. It is worth noting that mild health conditions are somehow related to the concept of sub-health (i.e., a state between perfect health and disease), which has recently become very popular in China [24]. In the field of Traditional Chinese Medicine (TCM), there is a particular emphasis on preventive treatment, that is, treating the disease before it’s caused or developed [40]. Fatigue and sleeping in the CHROME-G are items that exemplify the concept of sub-health, and these items have already shown the ability to report a higher number of milder health problems.

The convergent and divergent validity of the CHROME-G proved that the correlations between the CHROME-G and the EQ-5D-5L, SF-6Dv2 were mostly in line with the hypothesis of the study. However, the study also found a few correlations that deviated from the hypothesis. All of the insufficient correlations were found between the CHROME-G and the SF-6Dv2. The main reason may be the larger discrepancies in the descriptive systems of the two instruments, including item wording (e.g., “health status interferes social interaction” vs. “physical/emotional health interferes social activities”), level setting (based on severity vs. frequency), and recall period (past seven days vs. last four weeks). These discrepancies are likely to lead to differences in respondents’ understanding, and thus, to weaker correlations for the same concepts. Given these apparent differences between instruments, future users should be careful in their choice of instruments and cautious in exchanging results between different instruments.

All three instruments discriminated well among different health subgroups as expected, suggesting that they have good known-group validity. The CHROME-G and EQ-5D-5L tended to have significantly larger effect sizes than the SF-6Dv2 across extreme health levels (e.g. very good v.s. very poor in self-reported health states). However, this result differs from two previous studies, both of which compared the EQ-5D-5L and SF-6Dv2 in the Chinese general population. One study reported the effect size of SF-6Dv2 as 2.675 (95% CI 2.613, 2.737), which was higher than the effect size of EQ-5D-5L as 2.256 (95% CI 2.197, 2.315) between the “ ≥ 90” and “ < 65” EQ-VAS score groups [13]. Another study showed that the EQ-5D-5L and SF-6Dv2 had similar effect sizes (1.251 vs. 1.233) between the “no chronic conditions” and “ ≥ 3 conditions” groups [14]. The possible reason for this may be that previous studies have a healthier sample than this study (EQ-5D-5L utility: 0.939–0.947 in previous studies vs. 0.891 in this study; and SF-6Dv2 utility: 0.872–0.827 in previous studies vs. 0.697 in this study) and the SF-6Dv2 discriminates better in healthier people than the EQ-5D-5L. In addition, two previous studies were all face-to-face interviews and used utility values for effect size calculation, which may lead to differences in results.

We also found that the SF-6Dv2 had larger effect sizes than the EQ-5D-5L across milder health levels. That is, the CHROME-G, EQ-5D-5L, and SF-6Dv2 showed an effect size of 0.508 (95% CI 0.326, 0.690), 0.217 (95% CI 0.038, 0.397), 0.696 (95% CI 0.511, 0.879) respectively between the “very good” and “good” self-reported health states groups, and an effect size of 0.873 (95% CI 0.718, 1.028), 0.747 (95% CI 0.594, 0.901), 1.846 (95% CI 1.554, 2.137) respectively between the “ ≥ 90” and “80–89” EQ-VAS score groups in the EQ-5D-5L. It appears that the CHROME-G discriminates better than the EQ-5D-5L in healthier people and better than the SF-6Dv2 in unhealthier people. To clarify the known-group performance of the three instruments, more studies are needed in the future.

Although all the instruments possessed satisfactory test–retest reliability, the CHROME-G had a lower ICC than the EQ-5D-5L and SF-6Dv2. As calculated by the weighted Kappa statistics, the items pain, mood, fatigue, worry, memory, appetite, and sleeping in the CHROME-G showed more inconsistencies. These items tend to capture frequent health fluctuations and are, therefore, susceptible to change over time. Notably, many respondents reported changes in pain, fatigue, mood, or worry when completing the CHROME-G, whereas only a few respondents reported changes in similar items on the other two instruments. This may be mainly due to the different recall periods for the three instruments. With a shorter recall period of “today”, the EQ-5D-5L is likely to miss some health changes if respondents have not experienced new symptoms or are still staying in the old health state [41]. With a long recall period of “last four weeks”, respondents may focus on average levels of health states, which also leads to under-reporting of health change in the SF-6Dv2 [41]. In contrast, the CHROME-G employed a recall period of moderate length (last seven days). The difference of recall period may have an impact on the test–retest reliability of these instruments. For example, for the CHROME-G, although respondents reported no change in their health states between two surveys with a two-week interval, this does not necessarily imply that they had no change in pain between the past seven days of the first survey (before the interval) and the past seven days of the retest survey (the second week in the interval), particularly given that the pain item is a time-sensitive item. Besides, instruments have differences in the scope and meaning of some items (e.g., “pain” in the CHROME-G and “pain/discomfort” in the EQ-5D-5L), as well as in the level numbers and level wording; these differences may also potentially affect the agreement between measurement intervals. Another potential reason may be related to the questionnaire order and the interval between the two surveys. The test–retest reliability of the CHROME-G might be affected by it being the first instrument and the pain item being the first item in this instrument in both two surveys. Further research is warranted to continue to assess the test–retest reliability of the CHROME-G and to explore the underlying reasons why some items tend to change over time, especially for the poor performance for the pain item.

Several limitations of this study are summarized below. First, the online survey conducted in the study mainly included samples that could use mobile phones or computers and used only electronic questionnaires, all of which are different from the offline survey and are likely to have some impact on the results. We are planning to conduct an offline psychometric survey among the Chinese population and will compare the results between these two studies. Second, the study loosened the restrictions on changes in respondents’ health (allowing for a “slight change” in health and a 1-level difference in self-reported health status) to include more retest samples, which may underestimate the test–retest reliability of the three instruments. Third, the study didn't validate the responsiveness of the three instruments due to the difficulties in finding patients and tracking their health over time.

In the future, there is a need to collect more Chinese general population or patients’ responses to the CHROME-G and other existing Western-developed generic HRQoL instruments through offline surveys, and further compare their reliability, validity and responsiveness. In addition, it is worth comparing the CHROME-G with some generic quality of life (QoL) / Well-being instruments. Recent studies have shown that the ICEpop CAPability measure for adults (ICECAP-A) and the Recovering Quality of Life – Utility Index (ReQoL-UI) have different constructs from the EQ-5D and are more appropriate for measuring people’s mental health and well-being (a braoder QoL construct that goes beyond health) [42, 43]. The EQ Health and Wellbeing (EQ-HWB) also showed better known-group validity than the EQ-5D-5L in carers [44]. By identifing the measurement scope and properties through comparison with other instruments, the CHROME-G can further clarify its applicable population and contexts. This will also provide a sufficient and accurate measure selection basis for the associated economic evaluation studies.

## Conclusions

The CHROME-G was found to have good completion time, response distribution, convergent/discriminant validity, known-group validity, and test–retest reliability, and did not show any ceiling or floor effect in the general Chinese population. Compared with the EQ-5D-5L and SF-6Dv2, the CHROME-G showed better performance in terms of response distribution and known-group validity, which may help to measure the health states of the Chinese population more comprehensively and to discriminate the health level of the Chinese population more accurately. However, the performance of the test–retest reliability of the CHROME-G needs further attention and clarification.

## Supplementary Information

Supplementary Material 1

## Data Availability

The data are available only to the authors because we obtained informed consent from the respondents under that condition.
