# Supplementary material for: The generic version of China Health Related Outcomes Measures (CHROME-G): psychometric testing and comparative performance with the EQ-5D-5L and SF-6Dv2 among the Chinese general population
Source: BMC Public Health. 2024 Dec 18;24:3485. doi: 10.1186/s12889-024-20999-4 (PMC11656610; doi:10.1186/s12889-024-20999-4)
Supplement: Supplementary file 1 — Supplementary Material 1 [file 12889_2024_20999_MOESM1_ESM.docx]

Appendix Table 1

a. The minimum number for each quota in the first survey

| **Demographics** | **North** | **Northeast** | **East** | **Central** | **South** | **Southwest** | **Northwest** |
| --- | --- | --- | --- | --- | --- | --- | --- |
| **Age** |  |  |  |  |  |  |  |
| 18-29 | 23 | 15 | 57 | 30 | 23 | 28 | 13 |
| 30-39 | 23 | 16 | 59 | 31 | 23 | 29 | 14 |
| 40-49 | 24 | 16 | 59 | 32 | 24 | 30 | 14 |
| 50-59 | 23 | 15 | 57 | 31 | 23 | 29 | 13 |
| ≥60 | 27 | 18 | 68 | 36 | 27 | 34 | 16 |
| **Gender** |  |  |  |  |  |  |  |
| Male | 61 | 41 | 153 | 82 | 61 | 77 | 36 |
| Female | 59 | 39 | 147 | 78 | 59 | 73 | 34 |
| **Education** |  |  |  |  |  |  |  |
| Primary or lower | 32 | 22 | 81 | 43 | 32 | 40 | 19 |
| Junior high school | 47 | 31 | 117 | 62 | 47 | 59 | 27 |
| Senior high school | 21 | 14 | 52 | 28 | 21 | 26 | 12 |
| College or higher | 20 | 13 | 50 | 27 | 20 | 25 | 12 |
| **Residence** |  |  |  |  |  |  |  |
| Urban | 73 | 48 | 182 | 97 | 73 | 91 | 42 |
| Rural | 47 | 32 | 118 | 63 | 47 | 59 | 28 |

The quota sampling was used in this study, which five quotas, i.e., gender, age, education, urban/rural of residence, and region of residence, were pre-defined on the basis of their distribution in the Chinese general population. Statistics data of the Chinese general population were extracted from the *China Statistical Yearbook (2020)*. When the statistical scale of the original data was not calculated as the general population aged ≥ 18 years, the data were adjusted based on the proportion of the population of each age to the total population in this study. N/A indicates that data was not included in the public available data source.

b The minimum number for each quota in the retest survey

| **Demographics** | **The minimum number** |
| --- | --- |
| **Age** | 50 respondents each for 18-29, 30-39, 40-49, 50-59 and ≥60. |
| **Gender** | 50 respondents each for males and females. |
| **Education** | 50 respondents each for primary or lower, junior high school, senior high school and college or higher. |
| **Urban/rural of residence** | 50 respondents each for urban and rural. |
| **Region of residence** | 30 respondents each for Northeast and Northwest.  40 respondents each for North and South.  50 respondents each for Central and Southwest.  80 respondents for East. |

Appendix Table 2 Hypothesized conceptual correlations between the CHROME-G, EQ-5D-5L and SF-6Dv2

| **CHROME-G** | **EQ-5D-5L** | | | | | **SF-6Dv2** | | | | | |
| --- | --- | --- | --- | --- | --- | --- | --- | --- | --- | --- | --- |
|  | **Mobility** | **Self-care** | **Usual activities** | **Pain/dis-comfort** | **Anxiety/**  **depression** | **Physical functioning** | **Role limitation** | **Social functioning** | **Pain** | **Mental health** | **Vitality** |
| **Pain** |  |  |  | + |  |  |  |  | + |  |  |
| **Fatigue** |  |  |  |  |  |  |  |  |  |  | + |
| **Appetite** | - | - | - | - | - | - | - | - | - | - | - |
| **Vision** | - | - | - | - | - | - | - | - | - | - | - |
| **Hearing** | - | - | - | - | - | - | - | - | - | - | - |
| **Sleeping** | - | - | - | - | - | - | - | - | - | - | - |
| **Mobility** | + |  |  |  |  |  |  |  |  |  |  |
| **Daily activities** |  |  | + |  |  |  |  |  |  |  |  |
| **Mood** |  |  |  |  | + |  |  |  |  | + |  |
| **Worry** |  |  |  |  | + |  |  |  |  | + |  |
| **Memory** | - | - | - | - | - | - | - | - | - | - | - |
| **Social interaction** |  |  |  |  |  |  |  | + |  |  |  |

“+” indicates that the two items are conceptually similar and “-” indicates that the two items are conceptually different.

Appendix Table 3 Number of sample screened

|  | **Invited** | **Refused** | **Mid-round opt-out^a^** | **Quota exclusion^b^** | **Quality exclusion^c^** | **Included** |
| --- | --- | --- | --- | --- | --- | --- |
| **First survey** | 3078 | 211 | 108 | 1717 | 42 | 1000 |
| **Retest survey** | 594 | 15 | 26 | 164 | 11 | 378 |

^a^ “Mid-round opt-out” refers to the respondents who didn’t complete the survey and opted out.

^b^ “Quota exclusion” refers to the respondents who were excluded for noncompliance with the quota.

^c^ “Quality exclusion” refers to the respondents who were excluded for noncompliance with the quality control criteria.


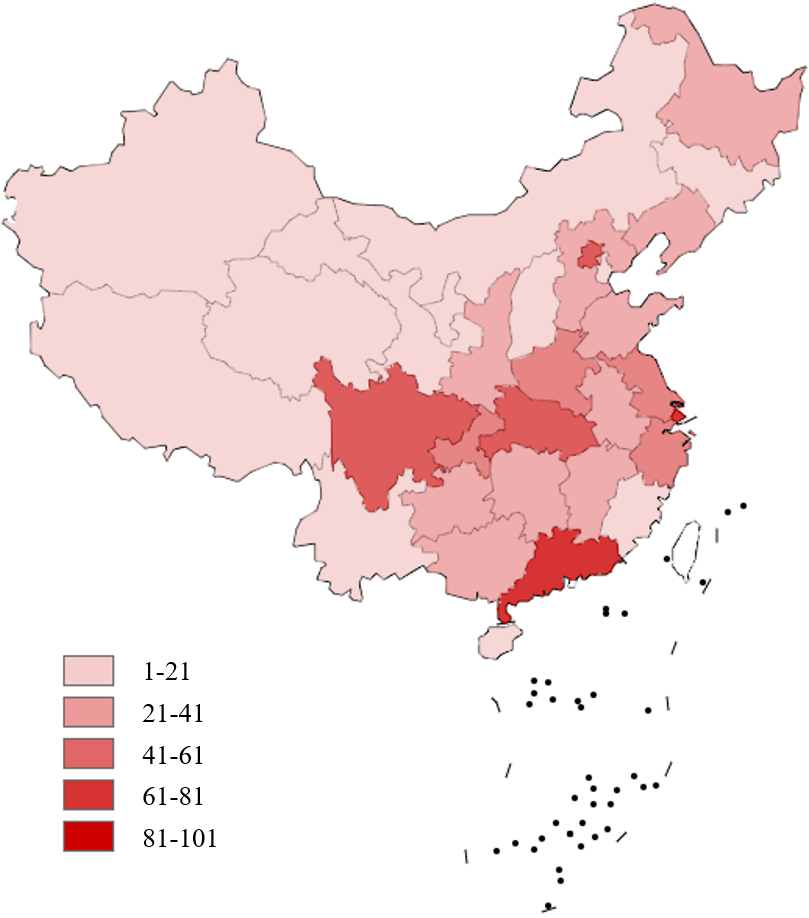

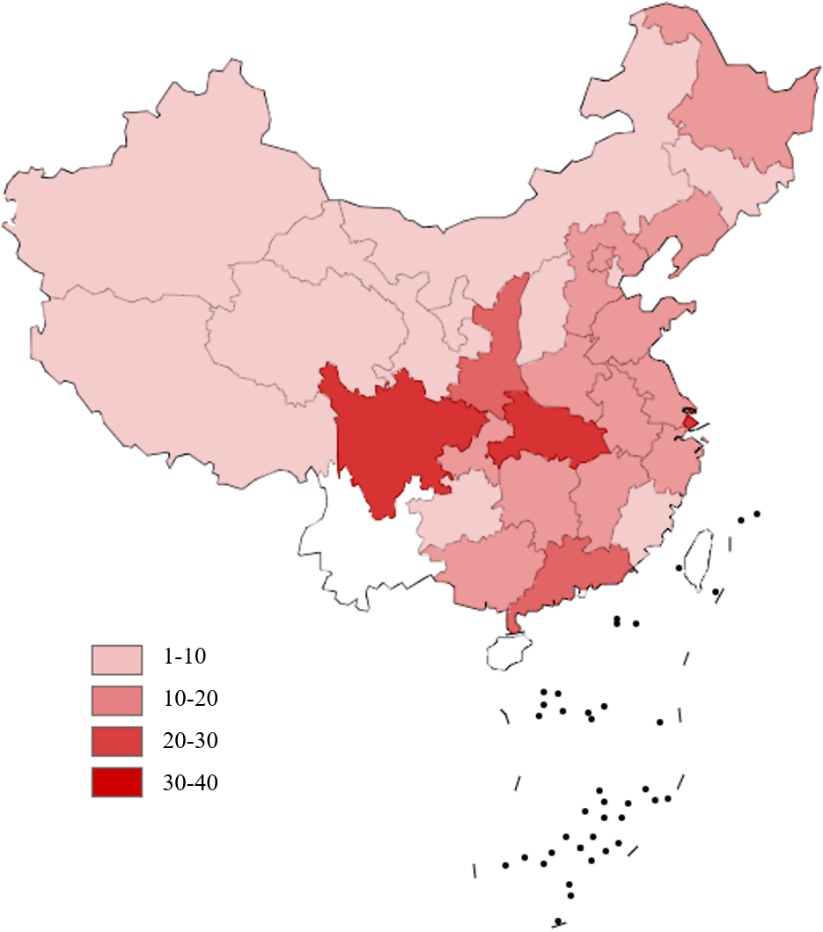


**a Region distribution for the first survey (N=1000) b Region distribution for the retest survey (N=378)**

**Appendix Figure 1 Geographic distribution of the sample**

**Appendix Table 4 The health utility value of sample**

|  | **Total sample (N=1000)**  **Mean ± SD** | **Retest sample (N=378)**  **Mean ± SD** |
| --- | --- | --- |
| **EQ-5D-5L utility value (based on Luo et al., 2017 value set)** | 0.891 **±** 0.143 | 0.913 **±** 0.096 |
| **EQ-VAS score in EQ-5D-5L** | 77.3 **±** 23.4 | 83.5 **±** 18.3 |
| **SF-6Dv2 utility value (based on Wu et al., 2021 value set)** | 0.697 **±** 0.195 | 0.719 **±** 0.149 |

Appendix Table 5 Feedback for the CHROME-G (N=1000)

|  | **Very easy** | **Easy** | **General** | **Difficult** | **Very difficult** |
| --- | --- | --- | --- | --- | --- |
| **Understanding** | 527  (52.7%) | 386  (38.6%) | 58  (5.8%) | 27  (2.7%) | 2  (0.2%) |
| **Completing** | 490  (49.0%) | 436  (43.6%) | 61  (6.1%) | 13  (1.3%) | 0  (0.0%) |

**a Level sum score distribution of the CHROME-G**

**b Level sum score distribution of the EQ-5D-5L**

**c Level sum score distribution of the SF-6Dv2**

**Appendix Figure 2 Level sum score distribution of the three instruments (N=1000)**
